# The Accessibility in the External Part of the TM5 of the Glutamate Transporter EAAT1 Is Conformationally Sensitive during the Transport Cycle

**DOI:** 10.1371/journal.pone.0030961

**Published:** 2012-01-23

**Authors:** Xiuping Zhang, Shaogang Qu

**Affiliations:** 1 China-America Cancer Research Institute, Guangdong Medical College, Dongguan, Guangdong, China; 2 Department of Immunology, Southern Medical University, Guangzhou, Guangdong, China; University of Cambridge, United Kingdom

## Abstract

**Background:**

Excitatory amino acid transporter 1 (EAAT1) is a glutamate transporter which is a key element in the termination of the synaptic actions of glutamate. It serves to keep the extracellular glutamate concentration below neurotoxic level. However the functional significance and the change of accessibility of residues in transmembrane domain (TM) 5 of the EAAT1 are not clear yet.

**Methodology/Principal Findings:**

We used cysteine mutagenesis with treatments with membrane-impermeable sulfhydryl reagent MTSET [(2-trimethylammonium) methanethiosulfonate] to investigate the change of accessibility of TM5. Cysteine mutants were introduced from position 291 to 300 of the cysteine-less version of EAAT1. We checked the activity and kinetic parameters of the mutants before and after treatments with MTSET, furthermore we analyzed the effect of the substrate and blocker on the inhibition of the cysteine mutants by MTSET. Inhibition of transport by MTSET was observed in the mutants L296C, I297C and G299C, while the activity of K300C got higher after exposure to MTSET. *V_max_* of L296C and G299C got lower while that of K300C got higher after treated by MTSET. The L296C, G299C, K300C single cysteine mutants showed a conformationally sensitive reactivity pattern. The sensitivity of L296C to MTSET was potentiated by glutamate and TBOA,but the sensitivity of G299C to MTSET was potentiated only by TBOA.

**Conclusions/Significance:**

All these facts suggest that the accessibility of some positions of the external part of the TM5 is conformationally sensitive during the transport cycle. Our results indicate that some residues of TM5 take part in the transport pathway during the transport cycle.

## Introduction

Glutamate is the major excitatory neurotransmitter in the central nervous system. The glutamate/neutral amino acid transporter family, also known as solute carrier family 1 (SLC1), includes many prokaryotic transporters as well as five excitatory amino acid transporters (EAAT) and two Na^+^-dependent neutral amino acid transporters. GLAST/EAAT1 [1], [2] and GLT1/EAAT2 [3] were cloned from rat while EAAC1/EAAT3 [4] was cloned from rabbit. EAAT4 [5] and EAAT5 [6] were both cloned from human. EAATs are the key elements in the termination of the synaptic actions of glutamate by uptaking glutamate from the synaptic cleft into cytoplasm [1], [2], [7]–[9]. So they serve to keep the extracellular glutamate concentration below neurotoxic level.

Glutamate transporters have a non-conventional topology. The amino-terminal half consists of six transmembrane domain segments, and the significantly more conserved carboxy-terminal half consists of two re-entrant loops and two transmembrane domains (TM7 and TM8) [10]–[12]. In addition, many residues of the carboxy-terminal half were implicated to be important for substrate and co-substrate binding and translocation [13], [14], [15]–[22]. A high-resolution crystal structure of a glutamate transporter homologue, Glt_Ph_, from the archeon *Pyrococcus horikoshii* was published [23], and it confirms and refines the topology as determined by functional studies [10], [11], [24], [25]. Moreover, the equivalent residues from the eukaryotic glutamate transporters, which are involved in potassium binding [13], [15], [26], sodium specificity [14], [16], [21], [22], and the ligand of the γ-carboxyl of glutamate [17], are located at the binding pocket of Glt_Ph_. Therefore, the Glt_Ph_ structure appears to be a good model for the study of its eukaryotic counterparts. It forms a trimer with a permeation pathway through each of the monomers. For the bacterial glutamate transporter and the neuronal glutamate transporter each monomer functions independently [27]–[31]. The first six transmembrane segments, which are less conserved, form a distorted ‘amino-terminal cylinder’ and provide all inter-protomer contacts, whereas the conserved transmembrane segments TM7 and TM8, together with hairpins HP1 and HP2, form the binding pocket [23]. HP2 is the external gate of the transporter and HP1 is the internal one. They alternately open so that the substrate-binding site can be accessed from extracellular or intracellular solution respectively [23], [32]–[35].

**Figure 1:**
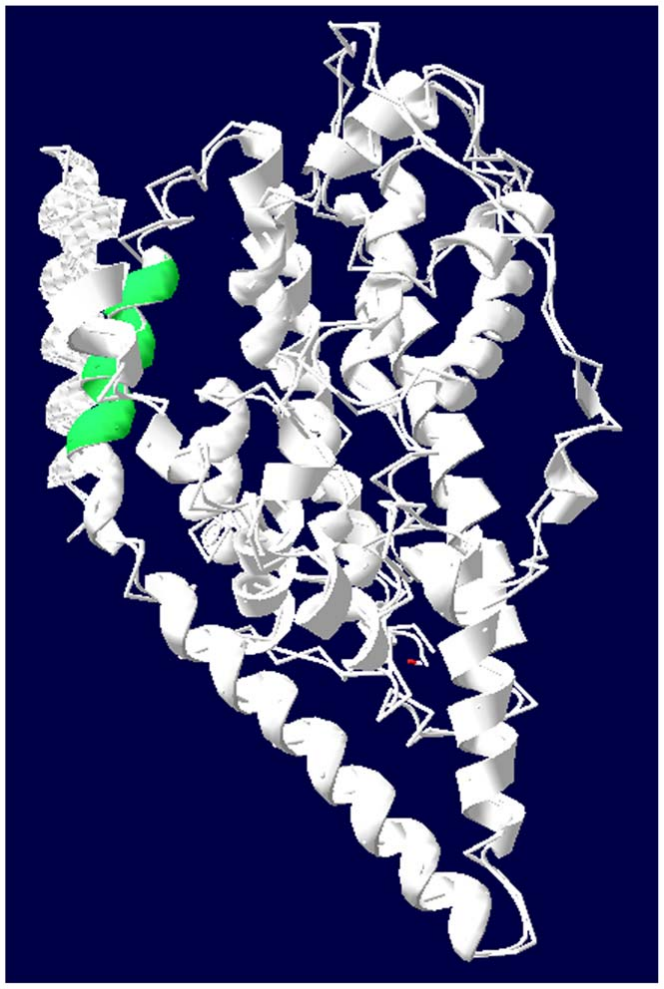
Structural model of Glt_ph_. The figure was prepared with the SPDB Viewer using the coordinates from PDB code 1XFH. The end of the TM5, from position 291 to 300, is colored green.

The trimeric interface of the glutamate transporter involving TMs 2, 4, and 5 is unchanged during transport [27]. To assess the functional significance of residues in TM5 of the cysteine-less version of EAAT1 (CL-EAAT1, in which the endogenous cysteines were replaced by serine or alanine, so that the interaction between the sulfhydryl reagents and endogenous cysteines is abolished), cysteine mutants were introduced from position 291 to 300, the end half of the Cysteine-less EAAT1 (Fig. 1). We checked the activity and kinetic parameters of the mutants before and after treatments with the membrane-impermeable sulfhydryl reagent MTSET [(2-trimethylammonium) methanethiosulfonate]; furthermore we also analyzed the effect of the substrate and blocker on the inhibition of cysteine mutants by MTSET. Our results indicate that the activity of some cysteine mutants was inhibited by MTSET, and glutamate and the glutamate's blocker, non-transportable glutamate analogue D,L-threo-β-benzyloxyaspartate (TBOA) can modulate this inhibitive effect. The data provides evidence that some residues of TM5 take part in the transport pathway during the transport cycle.

## Results

### Cysteine-scanning mutagenesis and impact of MTSET and MTSEA

**Figure 2:**
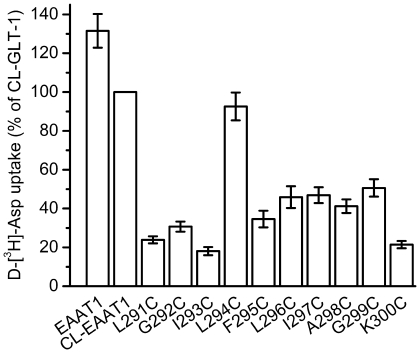
Transport activity of single cysteine mutants of TM5. The Cysteine-less EAAT1 and the TM5 mutants were expressed in HeLa cells. D-[^3^H]-aspartate uptake was measured as described under “Materials and methods”. Data are given as a percentage of Cysteine-less EAAT1 transport activity and are mean±S.E. of 3 to 4 experiments each performed in triplicates.

**Figure 3:**
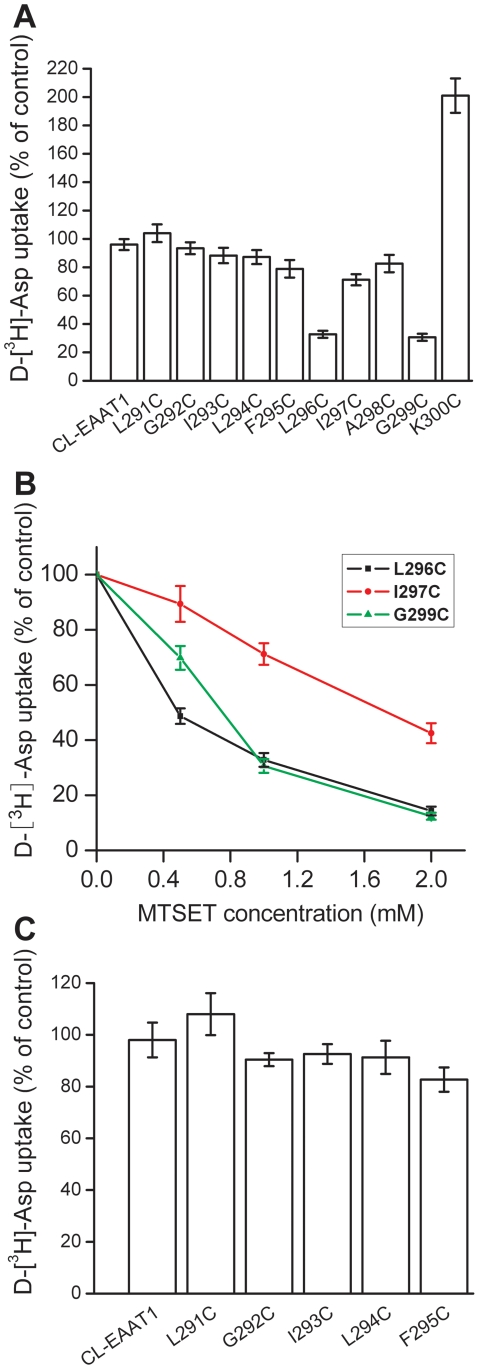
Effect of MTSET and MTSEA on uptake activity of single cysteine mutants of TM5. HeLa cells expressing single cysteine mutants or Cysteine-less EAAT1, were preincubated in NaCl-containing medium with sulfhydryl reagent for 5 min at room temperature, washed twice with choline chloride-containing solution, and subsequently D-[^3^H]-aspartate transport was assayed. Data represent percentage of the remaining uptake activity after incubation with sulfhydryl reagent relative to values obtained in the absence of sulfhydryl reagent. Values represent the mean ± S.E. of at least three separate experiments each performed in triplicate. (***A***) Effect of MTSET on transport activity of single cysteine mutants of TM5. The concentration of MTSET used in this study was 1.0 mM. (***B***) Effect of different concentrations of MTSET on transport activity of L296C, I297C and G299C. (***C***) Effect of MTSEA on transport activity of single cysteine mutants of TM5. The concentration of MTSEA used in this study was 2.5 mM.

Since the majority of TM5 is buried under the interior of the transporter, we only chose the end of the TM5, from position 291 to 300 to analyze the sensitivity of single cysteine mutants to MTSET. Cysteines were introduced one at a time into each of these positions of the Cysteine-less EAAT1. Cysteine-less EAAT1 has similar transport properties comparing with wild-type EAAT1, and it's an excellent background in which to introduce single cysteine mutants to study the function of the residues [36]. After transient expression of the Cysteine-less EAAT1 and the cysteine mutants in HeLa cells, transport of D-[^3^H]aspartate was measured. The activity of the cysteine mutants at these positions ranged from 18.1 to 92.6% of the Cysteine-less EAAT1 (Fig. 2). We expressed each mutant in HeLa cells and then measured the accumulation of radiolabeled D-aspartate before and after exposure to the membrane-impermeable sulfhydryl reagent MTSET. Transport by L296C, I297C, G299C single cysteine mutants was inhibited by 1.0 mM MTSET, while the activity of K300C got higher after exposure to MTSET (Fig. 3A). The sensitivity of L296C and G299C to MTSET was far greater than that of I297C, and the activity of these three mutants was inhibited by MTSET in a dose-dependent manner (Fig. 3B). An almost full inhibition of activity of L296C and G299C was observed when MTSET concentration was increased to 2.0 mM (Fig. 3B). With regard to the impact of the impermeant sulfhydryl reagent MTSET on activity, the end half of the TM5 can be divided in two regions: 1) the internal region, including from position 291 to 295, where none of the cysteine mutants are sensitive to MTSET; and 2) the external region, from position 296 to 300, where the mutants are impacted by MTSET with the exception of A298C (Fig. 3A). This suggests that the introduced cysteines at the external region are exposed from the extracellular side. Further more we also checked the cysteine mutants of the internal region with the membrane-permeable sulfhydryl reagent MTSEA [(2-aminoethyl) methanethiosulfonate] to know whether it is exposed from the intracellular side. MTSEA had no inhibitory effect on the activity of the cysteine mutants of the internal region (Fig. 3C), so the internal region isn't exposed from the intracellular side.

**Figure 4:**
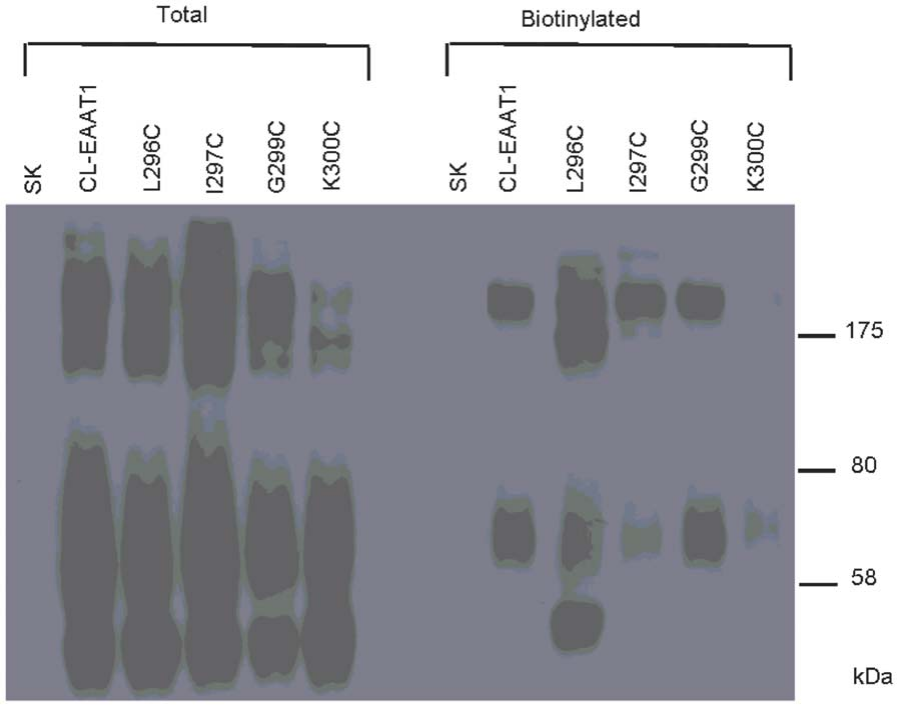
Cell surface biotinylation of Cysteine-less EAAT1 and mutants. HeLa cells, expressing Cysteine-less EAAT1 and the four indicated mutants, as well as HeLa cells transfected with the vector alone (SK), were biotinylated and processed as described in the Materials and Methods. The six left lanes show the total samples, and the six right lanes show the biotinylated samples. All samples were separated on the same SDS gel, transferred to nitrocellulose, and probed with the antibody to EAAT1. The positions of the molecular mass standards (in kDa) are indicated on the *right*. The experiment was repeated three times and a similar result was obtained. The result is representative of three independent experiments.

The biotinylation reagent only modifies proteins at the plasma membrane [37]. Surface biotinylation experiments showed that in the total samples of Cysteine-less EAAT1 and mutants, the major band has a mobility of 65–70 kDa, which represents mature monomeric forms of the transporter. The slower moving bands apparently represent dimeric/aggregate forms of the transporter (Fig. 4) [37], [38]. In the case of the biotinylated samples of Cysteine-less EAAT1 and mutants, the major band with mobility of 65–70 kDa were observed (Fig. 4). L296C and G299C were present on the plasma membrane at similar levels of Cysteine-less EAAT1 (Fig. 4). Scanning of the bands from the biotinylated samples from three different experiments indicates that intensity of the 65–70 kDa bands of I297C and K300C is statistically different from that of Cysteine-less EAAT1 (one-way ANOVA with post-hoc multiple comparison test, *p*<0.01). The percentage values of Cysteine-less EAAT1 are 78.2±11.6, 23.7±6.4, 94.8±5.3 and 11.3±2.5 for L296C, I297C, G299C and K300C, respectively. This experiment indicated that the lack of activity of I297C and K300C was due to defective targeting of these mutant transporters to the plasma membrane (Fig. 4). There are no transporter bands in the lanes labeled SK, which illustrated the specificity of the antibody (Fig. 4).

The kinetic parameters of glutamate were examined. In the presence or absence of MTSET, there is no significance difference between the *K_m_* values for L-glutamate of each mutant and that of Cysteine-less EAAT1 (Table 1). Furthermore the *K_m_* values of Cysteine-less EAAT1 and mutants were similar in the presence and absence of MTSET (Table 1). In contrast, after exposure to MTSET L296C and G299C have a significantly lower *V_max_* while K300C has a significantly higher one (Table 1).

**Table 1:** Kinetic parameters of CL-EAAT1 and single cysteine mutants.

	*Vmax*	*Km*	MTSET	
			*Vmax*	*Km*
CL-EAAT1	100	39.3±4.6	94.8±6.4	43.5±2.5
L296C	30.2±4.6	35.8±5.2	12.4±2.1*	37.9±4.7
I297C	28.1±2.7	42.7±4.1	22.7±4.3	41.2±3.6
G299C	25.6±1.9	36.9±2.6	11.9±2.8*	39.5±2.8
K300C	16.8±2.1	34.5±4.3	26.4±3.5*	38.4±3.2

*Km* (µM) and *Vmax* values were calculated for CL-EAAT1 and single cysteine mutants with and without MTSET preincubation. *Vmax* is expressed as a percentage of untreated CL-EAAT1. * denotes values that are significantly different after MTSET treatment. Values represent the mean ± S.E. of three independent experiments done in triplicate.

### Effect of transporter ligands on impact of TM5 single mutants by MTSET

For the cysteine mutants which are sensitive to MTSET, we checked the effects of transporter ligands on the sensitivity of single cysteine mutants to MTSET. From these experiments we tried to investigate the conformational change of TM5 in different stage of the transport cycle. The experiments were done in two stages. 1. Preincubation of HeLa cells expressing the single cysteine mutants with MTSET was done under various conditions reflecting different stages of the transporter (±Na, ±substrate, ±blocker). 2. After washing away the MTSET and substrate/blocker, the impact of the treatment was determined by measurement of transport.

**Figure 5:**
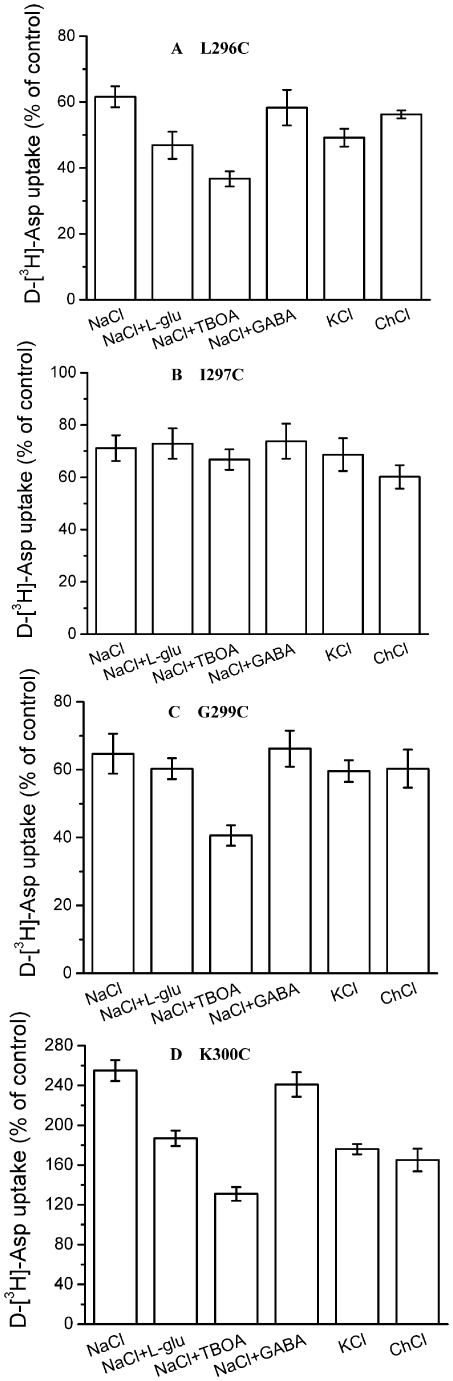
Effect of the composition of the external medium on the inhibition of single cysteine mutants by MTSET. HeLa cells expressing the single cysteine mutants L296C (***A***), I297C (***B***), G299C (***C***) or K300C (***D***), were preincubated for 5 min in the presence or absence of 0.2 (***A***), 1.0 (***B***), 0.6 (***C***) or 0.01 (***D***) mM MTSET. The indicated preincubation solutions contained NaCl, NaCl+1mM L-glutamate, NaCl+20 µM TBOA, NaCl+1mM GABA, KCl, choline chloride. Subsequently the cells were washed and D-[^3^H]-aspartate transport was measured as described under “Materials and methods”. Values are given as a percentage of control (preincubation without MTSET) and represent the mean ± S.E. of at least three different experiments done in triplicate.

For L296C, I297C and G299C, we applied an appropriate concentration of MTSET respectively to keep the transport activity of mutants around 60% of the control so that we could investigate the effects of the transporter substrate and blocker on the sensitivity of single cysteine mutants to MTSET (Fig. 5A, B and C). For I297C, the substrate L-glutamate and the glutamate's blocker TBOA had no effect on the inhibition of transport of I297C by MTSET, and we got the same result when NaCl was substituted by KCl (Fig. 5B). MTSET caused a stimulation of transport activity of K300C (201.2±12.1% of control, n = 3) (Fig. 3A). TBOA is expected to “lock” EAAT1 in an outward-facing conformation, increases the proportion of protein trapped in an outward-facing conformation, thereby increasing the aqueous accessibility from the extracellular side. Indeed, the inhibition of L296C and G299C by MTSET was significantly increased in the presence of TBOA (Fig. 5A and C). 36.7±2.3% and 40.6±3.0% activity remained after treatment with MTSET in TBOA for L296C and G299C respectively (Fig. 5A and C), while 61.6±3.2% and 64.7±5.9% activity remained after treatment with MTSET in NaCl for the two mutants respectively (n = 3) (Fig. 5A and C). When the sodium-containing medium was either supplemented with glutamate or replaced by potassium, it would increase the proportion of inward-facing transporters. In the presence of L-glutamate, a small but significant potentiation of the inhibition by MTSET was observed with L296C (46.9±4.1% of control in L-glutamate compared with 61.6±3.2% of control in NaCl, n = 3) (Fig. 5A), and the same was true for potassium (49.2±2.7% of control in KCl compared with 61.6±3.2% of control in NaCl, n = 3) (Fig. 5A). The potentiation was not observed with γ-aminobutyric acid (GABA), which are not substrates of EAAT1 (Fig. 5A), so it demonstrates the specificity of this effect by L-glutamate. On the other hand, L-glutamate did not have a significant effect on the inhibition of G299C by MTSET (Fig. 5C). The enhancement of K300C by MTSET in NaCl (255.4±10.5% of control, n = 3) was significantly decreased in the presence of L-glutamate, potassium and TBOA (187.2±7.8%, 176.1±5.1% and 131.3±6.9% of control respectively, n = 3) (Fig. 5D). Our results indicate that the accessibility of some positions of the end of the TM5 is conformationally sensitive.

## Discussion

The trimeric interface of the glutamate transporter involving TMs 2, 4, and 5 is rigid during transport [27]. The 3–4 loop of an archaeal glutamate transporter homolog plays an important role during the transport cycle, and cleaving the 3–4 loop results in the inactivation of the transporter [39]. Previously we have analyzed the proximity and functional significance of residues in TM5 and TM8 of the cysteine-less version of GLT-1. TM5 is in close proximity to TM8 in the glutamate transporter, and the spatial relationship between these domains is altered during transport [40]. From these findings we presume that TM5 maybe also take part in the substrate transport.

The introduced cysteines can react, through their sulfhydryl group, with a variety of sulfhydryl reagents. These reagents react in an aqueous environment since the sulfhydryl-group of the cysteine residue must be in its ionized form (Cysteine-S^−^). This reaction can lead to variable degrees of loss of function of the membrane protein, depending on the location of the cysteine and the importance of that position to the function of the protein. These sulfhydryl reagents react with the thiolate anion, and therefore, their reactivity appears to reflect accessibility of the introduced cysteine residues from the aqueous medium. These reagents include various MTS (methanethiosulfonate, CH_3_-SO_2_-S-R) reagents that differ in their size and charge. Some MTS reagents are membrane-permeable (MTSEA) and some are membrane-impermeable (MTSET).This difference in charge and bulk between the sulfhydryl reagents allows the investigation of specific positions changed into cysteine and allows the study of whether it is exposed from the extracellular or intracellular side.

When the amino acid residues 291–300 of the Cysteine-less EAAT1, located in the end of the TM5, all the mutants retain some level transport activity (Fig. 2). Three of ten of the mutants are inhibited by the membrane-impermeable sulfhydryl reagent MTSET (Fig. 3A). This suggests that these cysteines are exposed from the extracellular side. However, the end of the TM5 does not appear to form a completely open structure, because there are differences in sensitivity to MTSET between the different mutants (Fig. 3A). None of the cysteines introduced from position 291 to 295 are sensitive to MTSET, so these positions are not exposed from the external aqueous medium (Fig. 3A). MTSEA also had no inhibitory effect on the activity of these cysteine mutants (Fig. 3C). Therefore this region isn't exposed from the intracellular side. After exposure to MTSET the *K_m_* values of the mutants have no change but *V_max_* of L296C and G299C reduces, *V_max_* of K300C increases (Table 1). This data suggest that the reaction of L296C, G299C and MTSET rescinds the transport activity while the reaction of K300C and MTSET increases the transport activity. The reaction of the introduced cysteines and MTSET doesn't change the apparent affinity for glutamate, which means that the interaction of the mutants with L-glutamate isn't impaired, even though HeLa cells expressing mutants were treated by MTSET.

TBOA increases the proportion of protein trapped in an outward-facing conformation,thereby increasing the aqueous accessibility from the extracellular side. Indeed, the inhibition of L296C and G299C by MTSET was significantly increased in the presence of TBOA (Fig. 5A and C). There are three possibilities for this potentiation. One of the possibilities is that TBOA increases the aqueous accessibility of these positions from the extracellular aqueous medium. The other one is that after TBOA binding to the pocket, the external gate opens to the extracellular side which exposes the transport pathway to the extracellular solution [32], [41], so these positions might be expose to the transport pathway. Or a combination of these first two possibilities may be occurring. When the sodium-containing medium was either supplemented with glutamate or replaced by potassium, it would promote the formation of the inward-facing conformation with the external gate closed. In the presence of L-glutamate, a small but significant potentiation of the inhibition by MTSET was observed with L296C (Fig. 5A). On the other hand, L-glutamate did not have a significant effect on the inhibition of G299C by MTSET (Fig. 5C). These results suggest that G299C indeed is both exposed to the extracellular aqueous medium and the transport pathway in the presence of TBOA which keeps the external gate opening, so Gly-299 contact with the transport pathway. In this situation the extent of the exposure of G299C is maximal. In the presence of L-glutamate which keep the external gate closed G299C can't communicate the extracellular medium via the transport pathway, only directly exposes to the extracellular side, so the accessibility was decreased under conditions favoring the inward facing conformation of the transporter comparing with that in the outward facing conformation. In our earlier studies about the cysteine mutant G297C of GLT-1 (GLT-1 residue Gly-297 corresponds to Gly-299 of EAAT1) we also got the same results for the accessibility under different conditions [40]. As for L296C, L-glutamate and TBOA both cause a potentiation of the inhibition of transport by MTSET, but TBOA cause a potentiation to a higher extent (Fig. 5A). L296C might also be both exposed to the extracellular aqueous medium and the transport pathway in the presence of TBOA. In the presence of L-glutamate L296C can't communicate with the extracellular medium via the transport pathway, is only directly exposed to the extracellular side, so the accessibility was decreased compared with that in the presence of TBOA. However the transport of the substrate causes L296C to be much more directly exposed to the extracellular side, so the gap of the accessibility of L296C between in the presence of TBOA and L-glutamate was smaller than that of G299C.

Cysteines react, through their sulfhydryl group, with MTSET. This reaction can lead to variable degrees of inhibition of function of the membrane protein. However, in contrast to other mutants, MTSET increase the transport activity of K300C (Fig. 3A). It therefore appears that the reaction between K300C and MTSET can result in a complex conformational change, and this change is better for the substrate transport. The conformational change may include the compensatory shifting of other parts of the transporter. Now it is not clear what the nature of this conformational change is. The reactivity at this position was conformationally sensitive (Fig. 5D). The enhancement of K300C by MTSET was significantly decreased in the presence of L-glutamate and TBOA (Fig. 5D).

All these facts suggest that the accessibility of some positions of the external part of the TM5 is conformationally sensitive during the transport cycle. Our results indicate that some residues of TM5 take part in the transport pathway during the transport cycle.

## Materials and Methods

### Materials

D-[^3^H]-aspartate (11.3 Ci/mmol) and L-[^3^H]-glutamate (49.9 Ci/mmol) were purchased from PerkinElmer Inc. DL-TBOA was purchased from Tocris Bioscience. L-glutamate was purchased from Sigma. MTSET was purchased from Biotium Inc. DOTAP was purchased from Roche Molecular Biochemicals. DMEM, FCS, Trypsin(0.25%)-EDTA were purchased from Thermo Fisher Scientific Inc. Restriction enzymes were purchased from New England Biolabs. T4 Polynucleotide Kinase, T4 DNA Kinase, T4 DNA Polymerase, and T4 DNA Ligase were purchased from Roche. All other reagents were analytical grade.

### Generation and Subcloning of Mutants

The Cysteine-less EAAT1 in the vector pBluescript SK(–) (Stratagene) will be used as a parent for site-directed mutagenesis. The parent DNA was used to transform Escherichia coli CJ236 (dut–, ung–). From one of the transformants, single-stranded uracil-containing DNA was isolated upon growth in uridine-containing medium according to the standard protocol from Stratagene using helper phage R408. This yields the sense strand, and consequently mutagenic primers were designed to be antisense. Restriction enzymes *BsrG*I and *BstE*II were used to subclone the mutations into the construct containing Cysteine-less EAAT1 in the vector pBluescript SK(–). The subcloned DNA fragments were sequenced on both strands between the two restriction sites noted.

### Cell Growth and Expression

HeLa cancer cell line was purchased from ATCC (Manassas, VA). HeLa cells were cultured in Dulbecco's modified Eagle's medium (DMEM) supplemented with 10% fetal calf serum (FCS), 200 units/ml penicillin, 200 µg/ml streptomycin, and 2 mM glutamine. Heterologous expression of the wild type and mutant transporters was done as follows: HeLa cells plated on 24-well plates were infected with recombinant vaccinia/T7 virus vTF [42], and transfected with plasmid DNA encoding Cysteine-less EAAT1, mutants, or the vector pBluescript SK– alone using the transfection reagent DOTAP, as described [43]. Cells were incubated at 37°C for 16–20 hours.

### Cell Surface Biotinylation

After expression of transporters in HeLa cells (in 12-well plates), the medium was aspirated and the cells were washed twice with 2 ml/well of phosphate-buffered saline (PBS) containing 0.1 mM CaCl_2_ and 1 mM MgCl_2_ (PBS/CM). The cells were incubated on ice in 500 µl of 1.5 mg/mL NHS-SS-biotin (Pierce, Inc., Rockford, IL) in 20 mM HEPES, pH 9.0, 2 mM CaCl_2_, and 150 mM NaCl for two successive 20-min incubations. After labeling, the cells in each well were rinsed briefly with 1 ml of 100 mM glycine in PBS/CM and incubated in the same solution for 20 min on ice to quench unreacted NHS-SS-biotin. The cells were dissolved in 100 µl of lysis buffer consisting of 1% Triton X-100, 1% SDS, 150 mM NaCl, 5 mM EDTA, and 50 mM Tris-HCl (pH 7.5) by gentle shaking on ice for approximately 1 h until the cells were completely lysed, as assessed by light microscopy. The lysates from each well were then diluted by the addition of 900 µl of lysis buffer to reduce the concentration of SDS and clarified by centrifugation for 10 min at 16000g at 4 ^o^C. The biotinylated proteins were recovered from the supernatant solution by adding 100 µl of streptavidin-agarose beads (Pierce, Inc., Rockford, IL) and incubating 1 hour at 4 ^o^C with gentle agitation. The beads were washed three times with 1 ml of lysis buffer, twice with high-salt lysis buffer (lysis buffer containing 500 mM NaCl and only 0.1% Triton X-100), and once with 50 mM Tris-HCl (pH 7.5). The biotinylated proteins were finally eluted from the beads in 100 µl of SDS buffer according to the manufacturer's recommendation at 60°C for 10 min, 3 min on ice and then were centrifuged. Total samples were obtained by washing cells expressing Cysteine-less EAAT1 or mutantrs in 24-well plates twice with 1 ml/well of phosphate-buffered saline/CM, followed by lysis in SDS sample buffer, heating for 3 min at 60°C, and centrifugation. Samples were separated by SDS-polyacrylamide gel electrophoresis (10% gel) and transferred to nitrocellulose. The EAAT1 protein was detected with the antibody to EAAT1. Scanning and quantification of bands on the developed films was done using a Fluor-S™ MultiImager scanner with MultiAnalyst software, both from Bio-Rad.

### Transport

Transport of radioactive D-[^3^H]-aspartate or L-[^3^H]-glutamate was measured 16–20 hours post transfection. Plates were taken out. Wells were washed once with 1 ml of the choline solution (150 mM choline chloride, 5 mM KP_i_, pH 7.4, 0.5 mM MgSO_4_, and 0.3 mM CaCl_2_). Each well was then incubated for 10 min at room temperature with 200 µl of radioactive sodium uptake solution (150 mM NaCl, 5 mM KP_i_, pH 7.4, 0.5 mM MgSO_4_, and 0.3 mM CaCl_2_) which supplemented with 0.4 µCi of the tritium-labeled substrate. Uptake was stopped after 10 min by removing the transport solution and washing the cells twice in cold sodium uptake solution. HeLa cells were lyzed with SDS, and followed by scintillation counting.

### Inhibition Studies with Sulfhydryl Reagent

Before the transport measurements, the cells adhering to 24-well plates were washed with the choline solution. Each well was then incubated at room temperature with 200 µL of the preincubation medium containing MTSET or MTSEA (the different compositions are indicated in the figure legends). After 5 min, the medium was aspirated, and the cells were washed twice with 1 ml of the choline solution. Subsequently, each well was then incubated for 10 min at room temperature with 200 µl of radioactive sodium uptake solution. Then HeLa cells were lyzed with SDS, and followed by scintillation counting. Each experiment was performed at least three times. The concentration of MTSET chosen in the different experiments was optimized according to the mutants used.

### Kinetics

HeLa cells expressing Cysteine-less EAAT1 or mutants were measured for the ability to accumulate L-glutamate (200 µM;100 nM L-[^3^H]-glutamate, 199.9 µM unlabeled L-glutamate) as a function of time. Uptake kept linear for 10 min. HeLa cells were preincubated for 5 min in the presence or absence of 1.0 mM MTSET. Subsequently the cells were washed and incubated with 100 nM L-[^3^H]-glutamate in final unlabeled L-glutamate concentrations of 1, 5, 10, 50, 100, 250, 500 and 1000 µM for 10 min at room temperature. Then L-[^3^H]-glutamate transport was measured. Affinity constant (*K_m_*) and maximum velocity (*V_max_*) were determined by the Michaelis-Menten equation using KaleidaGraph (Synergy Software, Reading, PA). *V_max_* is expressed as a percent of that of untreated Cysteine-less EAAT1.
